# Influence of Insufficient Dataset Augmentation on IoU and Detection Threshold in CNN Training for Object Detection on Aerial Images

**DOI:** 10.3390/s22239080

**Published:** 2022-11-23

**Authors:** Arkadiusz Bożko, Leszek Ambroziak

**Affiliations:** Department of Robotics and Mechatronics, Mechanical Faculty, Bialystok University of Technology, Wiejska St. 45C, 15-351 Bialystok, Poland

**Keywords:** deep neural networks, image classification, data augmentation, object detection, unmanned aerial vehicle, aerial images

## Abstract

The objects and events detection tasks are being performed progressively often by robotic systems like unmanned aerial vehicles (UAV) or unmanned surface vehicles (USV). Autonomous operations and intelligent sensing are becoming standard in numerous scenarios such as supervision or even search and rescue (SAR) missions. The low cost of autonomous vehicles, vision sensors and portable computers allows the incorporation of the deep learning, mainly convolutional neural networks (CNN) in these solutions. Many systems meant for custom purposes rely on insufficient training datasets, what may cause a decrease of effectiveness. Moreover, the system’s accuracy is usually dependent on the returned bounding boxes highlighting the supposed targets. In desktop applications, precise localisation might not be particularly relevant; however, in real situations, with low visibility and non-optimal camera orientation, it becomes crucial. One of the solutions for dataset enhancement is its augmentation. The presented work is an attempt to evaluate the influence of the training images augmentation on the detection parameters important for the effectiveness of neural networks in the context of object detection. In this research, network appraisal relies on the detection confidence and bounding box prediction accuracy (IoU). All the applied image modifications were simple pattern and colour alterations. The obtained results imply that there is a measurable impact of the augmentation process on the localisation accuracy. It was concluded that a positive or negative influence is related to the complexity and variability of the objects classes.

## 1. Introduction

The Deep Neural Networks (DNN) are the most actively developed implementations in the machine learning field [1,2,3]. Their flexibility and plasticity for the input data genre make it an ideal solution for engineers, researchers and data analysts working in different areas all around the world. The computational power availability (provided by Graphics Processing Units acceleration and cloud clusters services) makes them highly affordable even for small companies and research facilities [4,5].

The main disadvantage of the DNNs is the necessity of performing the time-consuming training process; in some cases, it is possible to perform the fine tuning [6] of the network capable of solving a similar problem on the same input data [7]. However, it is not the solution for all the problems, even after the correct selection of the network architecture, node activation function and other parameters of the model, as the final performance is still limited by the quality of the training data delivered by the researcher. In many areas, the sufficient datasets might not be available or cannot be generated in the simulation, what indicates the need for collecting and labelling a new dataset. The data must be labelled in the large amount to prevent the overfitting [8]. The inability to collect the adequately diversified set of the images might be overcome by the artificial data augmentation [9,10]. The possible positive effect on assurance of the detection was postulated and confirmed many times for other visual [11], radar [12] and audio [13] data as well.

Dodge and Karam [14] evaluated four types of artificially generated distortions (blur, white Gaussian noise, jpeg conversion and contrast affection) that might occur on the digital photos. The model trained on the untreated data and tested on images with the modifications was poorly detecting the objects that were easily recognized by humans. Zhou et al. [15] tested the fine tuning and re-training as methods for overcoming this problem. Additionally, the works of Dodge and Karam [16] and Yuen and Zou [17] prove that this approach enhanced the performance; however, there might be an option for further improvement. The simple data augmentation techniques are widely used in many applications like traffic estimation [18] or leaf disease recognition [19].

The data augmentation techniques were also performed by the Convolutional Neural Network (CNN) structure alteration. The Dropband approach described by Yang et al. [20] crops the channels from the images and performs training on a newly generated set of deficient pictures. It is a method akin to the stochastic Dropout presented by Srivastava et al. [21], which randomly omits the set of neurons during the training. This approach is characterised by the high validation error deviation in comparison to deterministic Dropband. It was incorporated in numerous system architectures and used different applications such as medical diagnostics for brain structure [22] or lung diseases [23]. Both solutions can improve the DNN performance.

The other group of the augmentation techniques addresses the fact that one of the main purposes of the data augmentation is to improve the robustness for the distortions. This problem was also addressed with the development of methods based on machine learning algorithms. Their main function is to reduce the influence of the possible topological deformations. They are about the implementation of convolutional filters in the network structure. This method described by Bokar and Karam [24] still requires the training on the augmented data. The other approach presented by Lemley et al. [25] is based on the idea that the newly generated augmented data might be the result of a fusion of two other objects. The results of the research on that topic proved a high effectiveness in error rate reduction. The synthetical creation of the samples were also researched by Wong et al. [26], where the two approaches were compared: the data-warping that means creating the new samples within transformations in the data space and the synthetic over-sampling that means creating them in the feature space. The tests performed by the authors have showed that the first solution is more practical.

The general meaningfulness of the data augmentation was presented by Wang and Perez [11], where the reasonability of utilisation of the data augmentation for DNNs was concerned. The paper evaluates a possible performance gain in terms of additional time-consuming calculations and higher computing power requirements.

The research conducted by Volk et al. [27] on data augmentation showed clearly that the benefits in error rate minimization were significant and might be applied in real life scenarios. Nevertheless, the task of the aerial mapping and precise object detection inspired us to answer the question of whether it also improves the accuracy of the bounding boxes prediction.

### 1.1. Research Motivation

The presented research was conducted after the International Micro Air Vehicle (IMAV) competition, which was held in Melbourne in 2018. One of the tasks was the detection of the inflatable crocodile in the forest. Our team participated in that competition with a high score. The YOLOv3 (You Only Look Once version 3) network was used to detect the crocodile on the photos taken from UAV quadrotor flight at altitude of around 30m. There was prepared a dataset of over 3500 labelled photos for training, tests and validation sets. Having only one crocodile mockup and a handful of photos from the internet, it was decided to diversify the dataset by applying filters on some photos. The recall of the detected objects was oscillating around 75% despite the fact that the mockup on site was different than the one that was used to train the network. The other problem was the localisation of the object due to oversized bounding boxes. Those issues were consequences of the fact that the desired object class was unusual and a custom dataset had to be created, which was the most time consuming action during the competition preparations. It was realized that in some applications there might not be a feasible way to prepare the sufficient dataset. That is why it was decided to research the possible augmentation techniques for the insufficient and not numerous enough datasets. This made it faster to adopt the identification system to the search and to discover new objects without having to spend so much time preparing a large amount of input data.

Facing the previously reported problem of the YOLO framework—the inaccurate bounding boxes—it was decided to focus mainly on the IOU (Intersection Over Union) as a significant factor for the aerial applications of CNN image analysis systems. Knowledge about the influence of the data augmentation on the basic parameters like IoU and detection confidence might also be useful in the future research. Moreover, it is not researched as deeply as the augmentation effect on the learning rates and mAP [28]. The bounding box placement precision and size might be a particularly relevant in numerous applications beginning with the SAR (Search and Rescue) missions for drowning people, through fire surveillance to agricultural measurements. It is especially important when the images are not acquired from the downfacing camera, but from the low angled device. It might cause the significant relative localisation error due to the sensitivity of distance measurement in that case. In marine SAR tasks, the oversized bounding box in conjunction with the inaccurate live vest airdrop might lead to the mission failure. Quingqing et al. [29] engaged YOLOv3 to detect people in the water. For the object detection in training process the IoU threshold was set to only 0.1. It is motivated by the fact that when searching for drowning people, the false positive detections are much less undesirable then the false negatives. The rise of the average IoU allows us to ease the decision loop in such an application. In research by Yang et al. [30], the modified YOLOv4 was presented. It was analysing the image form USV to detect the body parts of the drowning people. The bounding boxes returned by the CNN are used to determine the position and pose of the target. These kinds of systems are also sensitive for IoU inaccuracy. In both mentioned papers, the datasets of the images were created by the researchers, and in Yang et al. [30], the dataset was augmented with simple operations; however, the impact of this operation was not examined. In Ribeiro et al. [31], the inaccuracy of the ships detection was overcome by the adoption of the real time instance segmentation method presented by Bolya et al. [32,33]. It allowed us to replace the bounding boxes with the masks obtained by the segmentation. In the process of network training, the synthetically enlarged dataset was used. The augmentation process was also performed in paper by Lei et al. [34], where the stationary underwater cameras were adapted for a swimming pool safety system. It compared YOLO versions from three to five and used stereo localisation of detected targets. In fire detection systems, an inaccurate bounding box might cause the overestimation or underestimation of the threat. The traditional handcrafted algorithms are being dominated by the CNN in fire detection applications that use the visual light cameras. Sharma et al. [35] used the VGG16 and Rasnet50 CNN architectures with additional fully connected layers were trained and tested on the custom image dataset. The modified Googlenet framework for this task was researched by Muhammad et al. [36]. The different CNN architectures were compared in terms of fire detection capabilities by Li and Zhao [37]. The bounding box detection might be crucial for the accuracy of the aerial agricultural measurements. The bounding box size might impact the crop damage estimations or counting trees in the area, Jintasuttisak et al. [38] created the image dataset with the fixed wing camera drone. It contained pictures of date palm trees. The detection ability was tested on the SSD300 and different yolo versions. The dominance of the YOLOv5 was proved in this application.

### 1.2. Paper Organisation

The remainder of the paper is organized in the following manner: Section 2 includes a description of a system architecture composed with UAV and camera, which provide aerial image data to the neural network and object detection algorithm. In Section 3, the image modifications used for data augmentations were described. Section 4 clearly and closely presents neural network architecture. Section 5 circumscribes the training process and dataset division. The coefficients used for the evaluation of the network were introduced in Section 6. Finally, Section 7 presents the results obtained in the research. Finally, Section 8 includes the conclusions taken from the conducted research.

## 2. System Architecture

**Figure 1:**
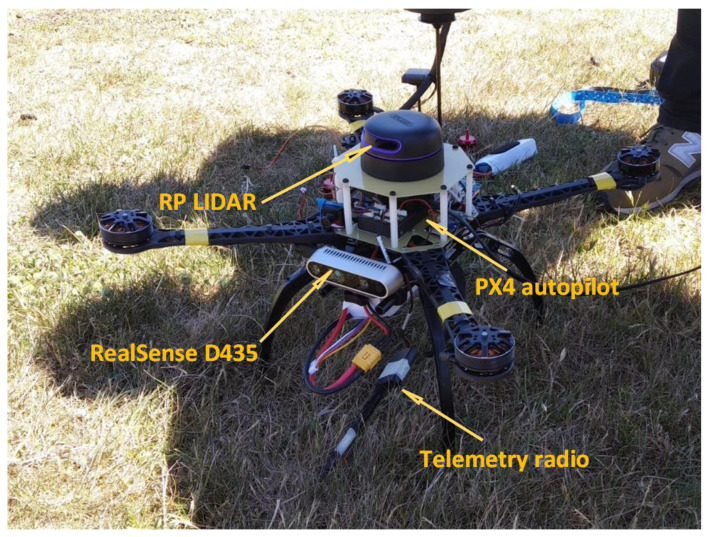
Quadrotor UAV used in the studies and during IMAV competitions.

**Figure 2:**
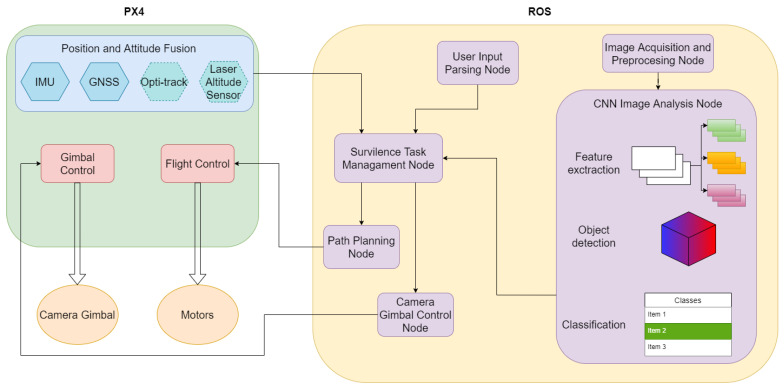
System architecture used in the studies.

**Figure 3:**
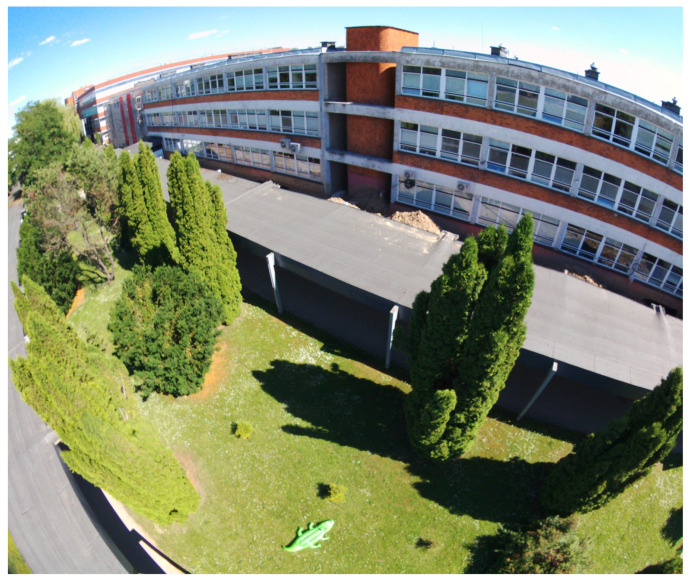
Example image taken from the drone camera as an input data to neural network system.

The system consists of two most important parts. The first part is a camera and unmanned aerial vehicle. The second is a computer with a working neural network and a visual image processing system. The UAV chosen for this task was a quadrotor platform (Figure 1) that was developed for testing many applications in recent years. In the current version, it is based on PX4 autopilot with real time software and ROS (Robotic Operating System) application working on a Nvidia Jetson family onboard computer. Additionally, there are other sensors on board, such as an IMU unit, GNSS receiver, RGBD sensors for the obstacle detection and avoidance system, and a laser range finder for precision altitude measuring. The mounted autopilot serves as a low level control device, which is responsible for UAV stabilisation, navigation and control of actuators (main motors, servos and gimbal motors). The ROS application is a set of independent programs called nodes that are working in a parallel mode. Each node is responsible for one task (e.g., taking and preparing data from camera, sending control commands, etc.). The data flow between nodes is based on a topic mechanism exploiting a TCP/IP stack. Neural analysis of the image is performed by the mentioned and proven YOLO architecture [39,40]. The simplified system architecture is presented in Figure 2. An important achievement is that the developed neural network has been tested and practically implemented on a low-power single-chip and non-efficient computer. An example of a photo taken from the camera during system tests is shown in Figure 3. The fisheye effect is visible on the picture. This type of distortion is often connected with the wide-angle cameras used on some drones and might theoretically affect neural analysis [41]. It later affected our choice of image modifications (see Section 3). The applications of DNN for image analysis in aerial tasks are limitless. However, they require that the training data consists of photos taken from a different perspective (down-facing camera), which also makes many open access datasets not fitted for critical purposes. Systems with such architecture are widely used in civil and military applications where an object and its position must be precisely recognized, but the type or the most important object’s features necessary for recognition are changing. For these reasons, the search has begun for quick opportunities to increase the input data set (images) and methods that would improve the quality of recognition and quickly adapt the system to search for new objects or similar objects with different characteristics (features).

## 3. Image Modifications

As shown in Section 2, the input data for the analysed object recognition system is a set of images of that object. Appropriate operations performed on the previously collected images may allow for an easy and quick increase in this input set. The possible data augmentation methods were described in by Mikołajczyk and Grochowski [42]. The three main approaches were indicated: traditional (simple shape and colour modifications), generative (with the GAN application [43] ) and the texture-transfer. The modifications applied in this research all belong to the first category and can be further divided into two groups. The first one contains the modifications of the colours in the image. These operations keep the topology of the image intact or they change it in the negligible level. The first used modification is a Red-Green-Blue (RGB colour channels) to grayscale (Y-single channel) transformation, what can be described as follows:

(1) Gray:Y←0.299·R+0.587·G+0.114·B.

This modification results with the image containing the information that is accentuating the shape features. The similar result might be obtained with other colour transformations, such as an image negative obtained by changing the source image $Img_{src}$ to destination $Img_{dst}$ form. The $R_{max}$, $G_{max}$ and $B_{max}$ are maximum values of the respective colour channels. It can be written as:

(2) $$Img_{dst}\left(R,B,G\right)=Img\left(R_{max},G_{max},B_{max}\right)-Img_{src}\left(R_{src},G_{src},B_{src}\right)$$

The next modification used is RGB channels remapping that can be realized by one of the following equations:

(3) $$Img_{dst}\left(R,B,G\right)\leftarrow \left\{\begin{array}{c} Img_{src}\left(R,G,B\right)\\ Img_{src}\left(B,G,R\right)\\ Img_{src}\left(B,R,G\right)\\ Img_{src}\left(G,B,R\right)\\ Img_{src}\left(G,R,B\right) \end{array}\right\}$$

To emphasise the attributes of the objects on the indistinct pictures the histogram equalization is used [44]. The next modification included into the transformations set is the contrast amplification described by the following equation:

(4) $$Img_{dst}\left(x,y\right)=\alpha \cdot Img_{src}\left(x,y\right)+\beta .$$

where *x* and *y* are pixel coordinates in the image and α>0 and β are the gain and bias, respectively. The last modification in the first group was a Gaussian blur application created with mask *G* of the size 3×3, 5×5 or 7×7

(5) $$G=\left[\begin{array}{cccc} g_{x=1,y=1} & . & . & g_{x=1,y=n}\\ . & . & \; & .\\ . & \; & . & .\\ g_{x=nn,y=1} & . & . & g_{x=n,y=n} \end{array}\right]$$

The matrix coefficients $g_{x,y}$ were calculated with equation based on the Gaussian distribution in the following manner:

(6) $$G\left(x,y\right)=\frac{1}{2\pi \sigma^{2}}e^{-\frac{x^{2}+y^{2}}{2\sigma^{2}}}$$

Whenever it was possible all the available parameters of the applied operations were randomly generated.

The second group of the applied modifications consist of the topological transformations. They keep the colours of the image unchanged. There first operation was a width/height proportion alteration. The second was an artificial perspective generation, where the input image is multiplied by the transformation matrix *M* defined as:

(7) $$M=\left[\begin{array}{ccc} M_{11} & M_{12} & M_{13}\\ M_{21} & M_{22} & M_{23}\\ M_{31} & M_{32} & 1 \end{array}\right]$$

where $M_{11},M_{12},M_{21},M_{22}$ form rotation matrix (around *x* and *y* axis), $M_{13},M_{23}$ represent output image translation, $M_{31}$ and $M_{32}$ combined is a projection vector. It is created from four coordinate pairs, resulting in the output image described as:

(8) $$\begin{array}{c} Img_{dst}\left(x^{\prime },y^{\prime }\right)=M*Img_{src}\left(x,y\right)\\ x^{\prime }=\frac{M_{11}x+M_{12}y+M_{13}}{M_{31}x+M_{32}y+M_{33}}\\ y^{\prime }=\frac{M_{21}x+M_{22}y+M_{23}}{M_{31}x+M_{32}y+M_{33}} \end{array}$$

The last topological image modification was generated with the application of the camera coefficients matrix *A* on the picture. The matrix usually used for camera calibration [45] is presented below:

(9) $$A=\left[\begin{array}{ccc} f_{mx} & s & c_{x}\\ 0 & f_{my} & c_{y}\\ 0 & 0 & 1 \end{array}\right]$$

**Figure 4:**
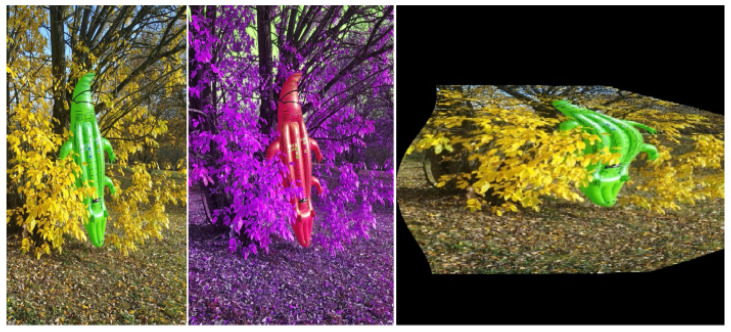
Example of an image modification. From left to right: original image, RGB channels remapping, random distortion matrix application (extreme example for better visibility).

In the matrix *A*, *f* stands for the focal length of the camera, *s* is the skew coefficient of the pixel grid, $c_{x}$ and $c_{y}$ are the principal point coordinates, and mx and my are scale factors in the *x* and *y* axes. Similarly, as in the previous group of the parameters as proportion factors, perspective orientation and the distortion matrix coefficients were randomly generated. A sample modification of the image is presented in Figure 4.

The equation:

(10) $$D_{n}=D_{o}+\bar{D_{o}}.$$

describes the new training set. Variables $D_{n}$ and $D_{o}$ are, respectively, corresponding to the new set and the old set. The $\bar{D_{o}}$ represents the image set derived from the $D_{o}$ after applying the described modifications.

## 4. Network Architecture

**Figure 5:**
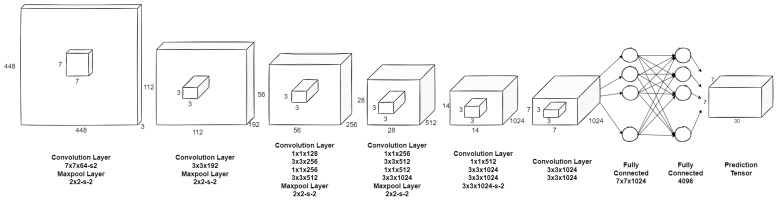
Original YOLO network architecture schematic.

The YOLO convolutional neural network in relation to prior works presents an unusual approach to the object detection task. Instead of harnessing the classifiers to detect objects, it treats object detection as a “regression problem to spatially separated bounding boxes and associated class probabilities” [46]. It utilises multiple image analysis steps in a single network. It is characterised by the lack of sliding window detectors or Region of Interest (RoI) detectors (region proposal-based techniques). The network pipeline is significantly simplified in relation to many state of the art solutions. This approach makes the training process reasonably fast. It also looks at the features in a global context, which reduces the number of background errors. The YOLO network divides the input image into an S × S grid. Each of the grid cells predicts the presence and probability of the bounding boxes with its parameters (size and position of the box). Simultaneously, the occurrence probabilities of the object of the known classes are calculated for each cell, regardless of the number of predicted overlapping bounding boxes. As a result, the class probability map is produced. Then, the MLP (Multi Layer Perceptron) aggregates the data and returns the output in the form of list of bounding boxes with their size, position, class and confidence. The original network has 24 convolutional layers (9 in lightweight version) and two fully connected layers. The kernel size is 3 × 3. The input image is supplied to the network with a resolution of 448 × 448 pixels (for classification, the pretrained convolutional layers were feed with 224 × 224 pixel images). The schematic of the network is presented in Figure 5.

The activation function used in the neurons is a version of leaky rectified linear activation (ReLU) (11).

(11) $$\phi \left(x\right)=\left\{\begin{array}{cc} x & \mathrm{for}\;x>0\\ 0.1x & \;\mathrm{for}\;x\;\leq \;0\;\; \end{array}\right.$$

**Figure 6:**
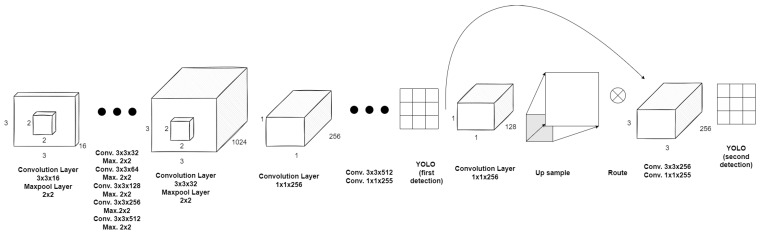
YOLOv3 tiny version network architecture schematic.

In YOLOv2 architecture, many improvements were introduced helping achieve higher mAP (mean Average Precision) and IOU. The convolutional layers were batch normalised and the classifier was pretrained with a higher resolution in first 10 epochs, and those modifications both improve the mAP network score. The fully connected layers were removed and replaced with the anchor boxes for the purpose of the bounding boxes prediction. The lack of MLP on output allowed the multi-scale training. The input size is randomly changed after each 10 batches, varying in size from 320 × 320 to 608 × 608 pixels. This flexibility helps balance the trade-offs in terms of detection/classification and processing speed. This architecture was used to train network YOLO9000, which is capable of predicting 9000 different classes after the joint training algorithm performed on the ImageNet and COCO (Common Objects in Context) datasets [47]. In the YOLO version 3 presented in [48], the high framerate in real time detections was sacrificed for a further boost of the detection accuracy. It shifts the number of convolutional layers and introduced residual blocks, up sampling and skip connections. The feature extraction training is performed on the Darknet-53 architecture with 53 convolutional layers. The class prediction is made on a full version of the network with 107 layers (75 convolutional, 23 shortcut, 2 up sample, 4 route and 3 yolo layers). It utilises the prediction across scales. The objects are detected by ‘yolo’ layers (anchor concept implementation) on images with the following scales: 320 × 320, 416 × 416 and 608 × 608 pixels. It provides the network with the capability of size invariant detection sensitivity. The lightweight version called YOLOv3-tiny derives from the described generations. It provides reasonable performance with the minimal workload. It enables efficient real time image analysis using small onboard computers for unmanned aerial vehicles. For simple aerial applications with a low variety of classes detected, it is considered to be the optimal solution. Using prediction across scales (2 levels), anchor concept, 13 convolutional and 6 maxpool layers it provides more than enough to produce repeatable results in short time. Although it might restrict flight altitude span, YOLOv3-tiny was chosen as the research tool. The network schematic is presented in the Figure 6. It is well a proven and extensively tested version. The literature studies show that in embedded applications like UAV [49], the rate of the object detection with YOLOv3 architecture is comparable or faster than with the newer versions. Even in the latest articles about object detection, YOLOv3-tiny is still often used [50,51].

However, being more sensitive to inaccurate localisation of the features and objects it is more robust for false positive output [46]. Due to some advantages over other popular CNN architectures (lightweight and suitable for real time [52,53,54], many research groups develop further network versions [55,56]. Some of them were created in order to use them for UAV images analysis [39].

## 5. DNN Training

For the purpose of this research, three image sets were prepared. The first of them is considered as an original data. It contains objects of seven classes: hammers, pliers, screwdrivers, cups, keyboards, computer mouses, and inflatable crocodiles. Due to the basic assumption of the research, dataset must be small and easy to collect. Our image library contained only just over 500 labelled objects in total. The small size of the training image dataset was chosen due to the research assumptions described in Section 1.1. It results in the lower quality performance; however, it also makes the potential gain after the modification being higher and easier to indicate. The second set was created after extending the original data with the modified copies of the photos. The transformations considered as simple were used in this step. Photos in this step were modified using following equations (in brackets) from section Image Modifications: RGB to grayscale conversion (1), channels swap (3), image negative (2), Gaussian blur (6), (5) and proportion modification. Each photo appeared in six modified copies (original image plus one for each transformation). For the last dataset, all the modification from the previous step were used. Additionally, the collection of the transformations was extended with the histogram modification, contrast gain (4), perspective generation (8), (7) and distortion application (9). The total number of samples created from the single image in this step was 10.

All image sets were used to train neural networks, which were named NN1 (trained on the original dataset), NN2 (first modified dataset) and NN3 (second modification of the dataset). Each of the training sessions was terminated after 17,000 epochs, which corresponded to the 24 h training cycle on the CUDA (Compute Unified Device Architecture) enabled workstation. With this approach during each training session, every object labelled on the photos was processed with the constant average number of iterations. However, in series two and three, the objects have a great chance to be modified in various iterations. Combination of this characteristics with the random nature of the modifications is expected to prevent the neural network from overfitting. The models were trained using the YOLOv3-tiny network implemented with the Darknet framework. All the training sessions were performed with the same parameters.

## 6. Evaluation of the Augmentation Influence

For the evaluation of the augmentation influence, two coefficients were used. Having the small dataset as it was postulated in the research motivation section, the high performance was not expected. For this reason, to evaluate the augmentation influence, the fundamental low-level measures were made, which are usually critical in terms of popular CNN performance metrics [57].

The first factor was detection confidence returned by the model for each found bounding rectangle overlaying ground-truth label (true positive). The confidence returned as a percentage value does not correspond to the statistical chance of the object being correctly detected and is dependant on the network architecture. However the arbitrary chosen confidence threshold influences detector performance. Higher average confidence for true positive detections might allow for easier finding of the network optimal threshold [58].

**Figure 7:**
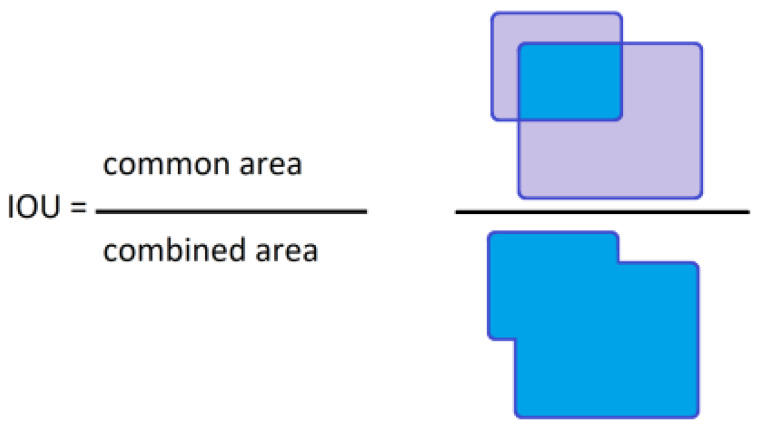
Schematic of the IOU calculation.

The second one, IOU—Intersection Over Union—factor, was calculated after the comparison of the bounding boxes from the label attached to the image in the validation set and the bounding box generated on the output of the trained network. In the numerator there is a common area of the two rectangular boxes, and in the denominator there is an area of the two boxes combined. The IOU factor is fitted in the range <0;1>. Figure 7 represents the idea of this calculation.

## 7. Results

### 7.1. Detection Confidence

**Figure 8:**
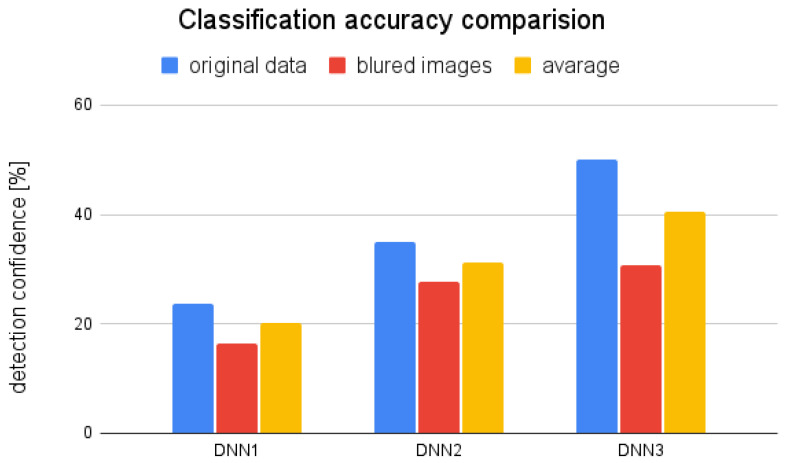
Average confidence returned by models trained on the different datasets.

The evaluation of the networks performance was divided into two steps. First, the network detection confidence was measured. The test set was composed of 25 objects of each class. Figure 8 presents the results of the detection confidence comparison between three trained models. As it can be seen the network NN1 trained on the original data provided low quality results even it was performing well on the validation data. It is an obvious result of the overfitting caused by the insufficient size of the training dataset. The effect that was naturally inherited by the two remaining networks was firmly constrained by addition of the augmented data samples. The effect was visible particularly on the unmodified images and was less evident on the blurred data, as in networks NN2 and NN3 trained on the augmented datasets there was the same number of images with that particular distortion. The enlargement of the data set will reduce the relative gain in the models trained on the properly numerous datasets.

### 7.2. Bounding Rectangle Accuracy

The performance of the trained networks in terms of the bounding rectangle assignment accuracy was measured after every thousand epochs. The value of the IOU coefficient was measured on the test set containing 25 objects of each classes. The values on the plots are the average of these detections. The object was considered as correctly classified only if the detection confidence was at least 50%.

**Figure 9:**
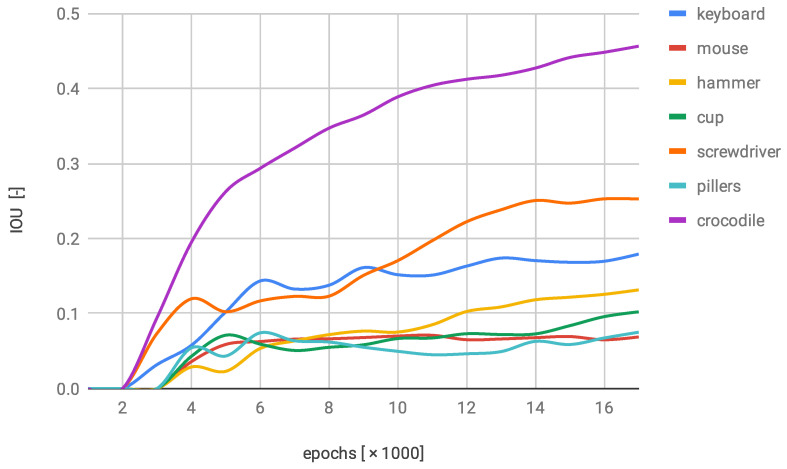
IOU changes over training progress on original dataset.

In Figure 9, there is a plot representing the performance of the NN1. It is typical for this network that there is one class of objects with the overwhelmingly exceeding effectiveness. Other classes were barely detected by the network. This is probably an implication of the insufficient number of different object instances in the dataset.

**Figure 10:**
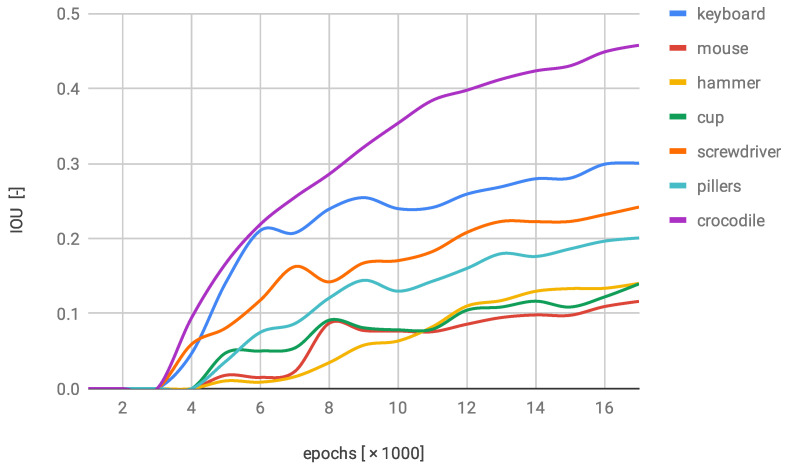
IOU changes over training progress on first modified dataset.

The second examined network NN2 trained on the modified dataset brought a minor enchantment to the performance. Six out of the seven classes were labelled with the higher accuracy. However, only two of them reached the evident progression (keyboards and pliers). One of the classes achieved a small regress. It might have been caused by the fact that the object (screwdriver) is relatively simple and any change might disturb the characteristics too heavily (Figure 10). All classes were marked with the average IOU factor higher than 0.1 (in the first network, two of the classes did not exceed that threshold).

**Figure 11:**
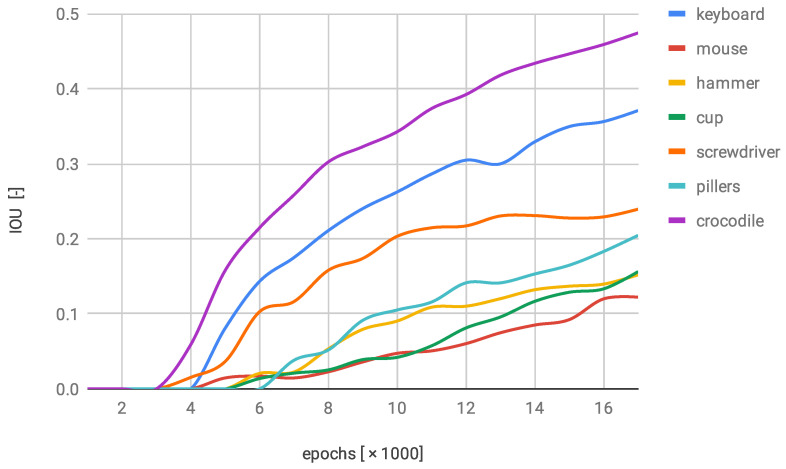
IOU changes over training progress on second modified dataset.

After the evaluation of the NN3, there were still three classes that have not been labelled with the IOU coefficient at least 0.2, although the rest of the objects were discovered with higher precision rates. The gradual augmentation can be seen in Figure 11. This was the first network performance validation where two data series exceeded the 0.35 of the IOU factor. It is noticeable that after the last epoch all series overstep the 0.1 again.

### 7.3. Detection Examples

**Figure 12:**
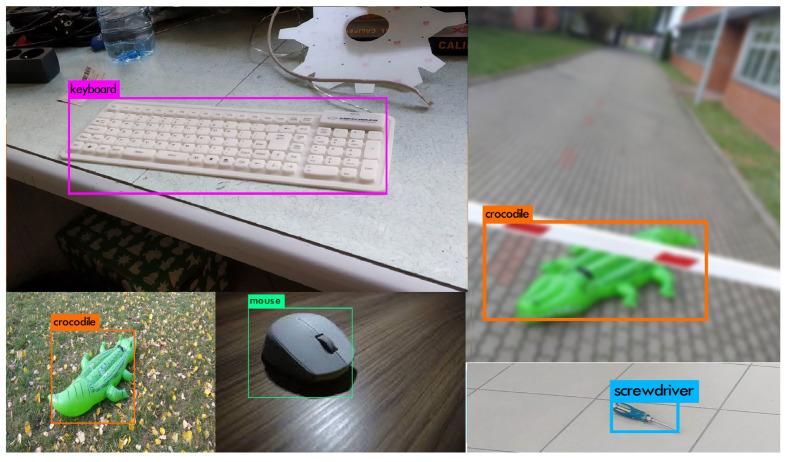
Examples of correct detections.

In Figure 12, some examples of the correct bounding box predictions are shown. Despite the fact that some labels generated by the network might seem almost perfect, their IOU coefficient is in range from 0.67 (computer mouse) up to 0.94 (keyboard). Achieving this level of performance might still be considered as acceptable.

**Figure 13:**
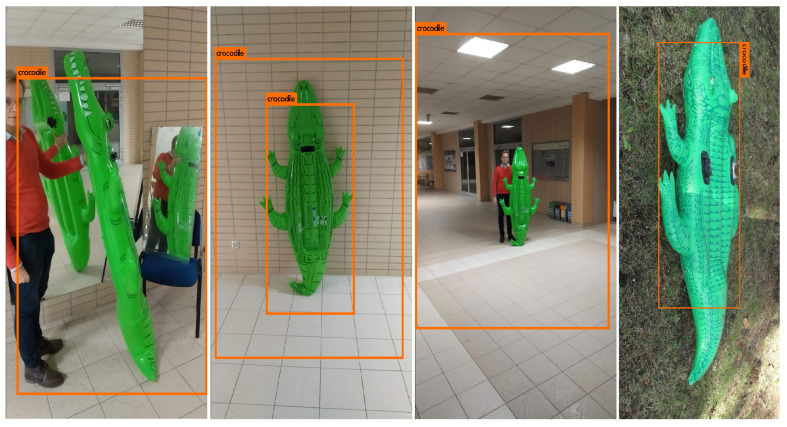
Examples of incorrect detections.

However, not all detections were correct. In some cases, the trained models were suffering from different issues. In Figure 13, there are examples of some common faults. The image with multiple crocodiles was classified as a single object, which caused the significant IOU reduction. What is more, this type of error is omitting some information that should be returned by the network. There is also an example of a too-small rectangle (image on the right) and a too-big one (third image). This leads to the reduction of DNN evaluation in terms of IOU. The next meaningful defect is the multiple detection of the single object.

### 7.4. Trained Models Comparison

**Figure 14:**
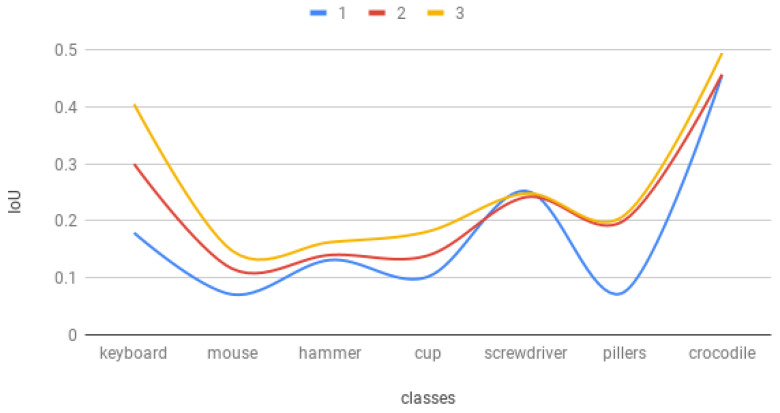
Comparison of the results of the research after training sessions on three prepared datasets (1-original, 2-first modification, 3-second modification).

The performance in the succeeding research stages is placed in Figure 14. Three lines: blue, red and yellow represent, respectively, the performance of the models NN1, NN2 and NN3. There can be indicated a regularity on the graph. Each next training session produced better results than the previous one. The only point where it is not occurring is the detection of the screwdrivers. For this particular class, the best results were achieved with the network trained on the original dataset. Modifications that affect the shape of the item could not immunize the system from the disruptions or glitches, instead it only diminishes the sensitivity of the actual pattern.

The second network reached the highest absolute gain in comparison to the first one in pliers class detection (12%). However, the performance growth for this class was barely noticeable for the last network, where the most articulated boost was related with the keyboard class. Three classes of objects achieved relative growth higher than 100% in the network trained on the second modification dataset. It is mostly caused by the low initial IOU value for the network trained on the original images. However, the average absolute gain in comparison to NN1 is, respectively, 4.6 and 8.1 for NN2 and NN3. The detailed performance progression can be found in Table 1.

### 7.5. The Detection Confidence and IOU Correlation

**Figure 15:**
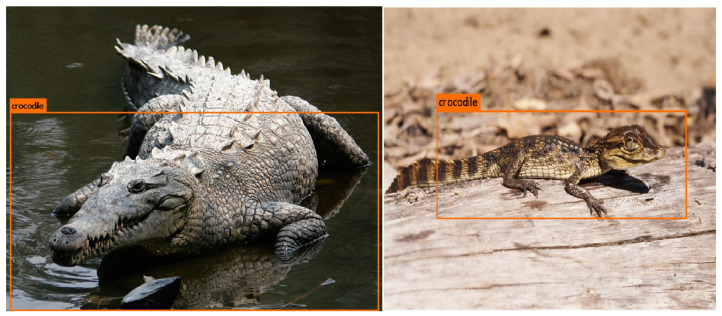
Occurrences of the real crocodile classification [59,60].

The last network training (NN3) was not cancelled after 17,000 epochs, but instead it was allowed to get up to 40,000 epochs. The network performance was naturally significantly better according to the results that were presented in previous sections. What is interesting is that it was the first network tested that was able to detect the real crocodile on the image provided (Figure 15) (the accuracy was negligible in most cases; however, it is appealing because of the fact that no photos of real reptiles were used in the training process).

**Figure 16:**
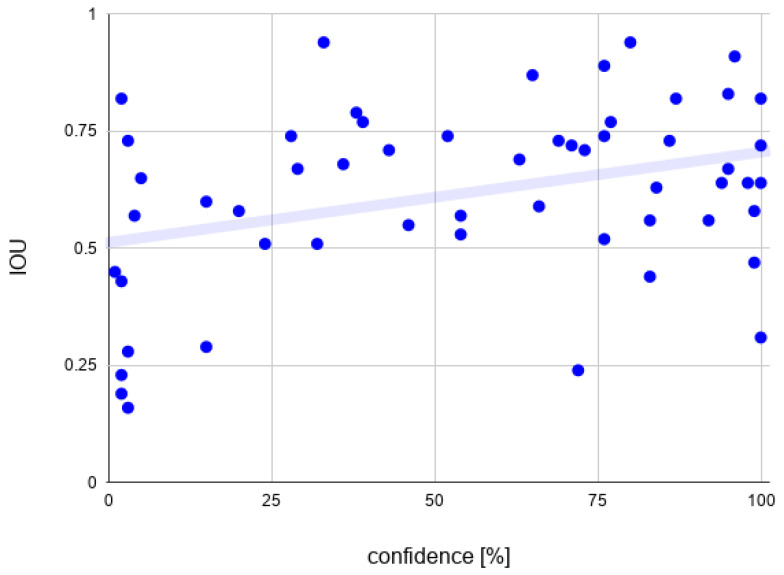
Data points used to calculate the correlation.

On this network, the Pearson’s correlation coefficient was calculated for the detection confidence and IOU. In Figure 16, the map of the 50 arbitrary chosen data points is presented. Only the correct classifications were taken into account (correct classification was considered for the objects that had non-zero IOU and there was no higher score for the incorrect match). The value of the correlation coefficient equals 0.37, which means that despite the fact that both parameters used to measure network performance are indicating the positive influence of the data augmentation on DNN performance, they are not strongly related. The progression in detection confidence does not necessarily indicate the IOU accuracy betterment for the particular object class and vice versa.

## 8. Conclusions

The results obtained in this research prove that the detection parameters quality of the neural networks trained on the deficient training datasets might be improved with the application of simple augmentation techniques. The increase of the detection confidence for the true positive instances might cause easier tuning of the detection threshold. Even the small enhancement of the IoU parameter perceived in the networks trained on the augmented datasets is a valuable gain in terms of object localisation. This might have a significant influence on the precision of target placement in reference coordinates, when applied in search and rescue aerial missions and other UAV assignments.

The effects of the particular modifications might differ depending on the classified objects complexity. The simple featured objects like screwdriver, pen or balls might be sensitive for topological modifications even if they are of low intensity. The more complex objects that might occur in different poses, like animals or legged robots are expected to gain benefits from shape changing augmentations. The analogical situation can be recognised in case of colour modifications. Having this in mind, while preparing the data augmentation, the researcher should be aware what is the exact purpose of the needed network and how variable are the features of the trained classes.

The performance improvement accomplished in networks trained on the augmented datasets shows that both, the detection confidence and the IOU of the detections are affected by the data augmentation. However, the low Person’s correlation coefficient value, points that the even the nearly full detection confidence is not a sufficient evidence for the bounding box prediction accuracy. The results show that a further research need to be done in this area. The most important issue is to define the influence of the particular modifications on the performance of the neural network. This research needs to be done on the extended dataset and networks with significantly lower error rates.

## Figures and Tables

**Table 1:** The absolute and relative gain for each class of the objects after first and second dataset modification [%].

		Classes							
**Gain**	**Modification**	**1**	**2**	**3**	**4**	**5**	**6**	**7**	**Average**
absolute	DNN2	12.1	4.5	0.8	3.7	−1.0	12.5	0.1	4.6
	DNN3	22.6	7.6	3.1	7.9	−0.4	13.4	2.5	8.1
relative	DNN2	67.5	63.7	6.6	36.4	−4.3	167	0.2	48.2
	DNN3	126	108	23.7	77.6	−1.7	178	5.6	74.0

Clsses indexes: 1—keyboard, 2—mouse, 3—hammer, 4—cup, 5—screwdriver, 6—pliers, 7—crocodile.

## Data Availability

Not applicable.
